# Whole genome sequencing reveals antimicrobial resistance determinants (AMR genes) of *Salmonella enterica* recovered from raw chicken and ready‐to‐eat leaves imported into England between 2014 and 2019

**DOI:** 10.1111/jam.15728

**Published:** 2022-08-05

**Authors:** Nicola Davies, Frieda Jørgensen, Caroline Willis, Jim McLauchlin, Marie Anne Chattaway

**Affiliations:** ^1^ Gastrointestinal Bacteria Reference Unit (GBRU) UK Health Security Agency London UK; ^2^ Division of Infection and Immunity University College London London UK; ^3^ Food Water and Environmental Microbiology Laboratory Porton UK Health Security Agency Salisbury UK; ^4^ Food Water and Environmental Microbiology Services UK Health Security Agency London UK

**Keywords:** chromosomal mutations and antimicrobial resistance genes, imported food, monitoring, *Salmonella*, whole genome sequencing

## Abstract

**Aims:**

To compare the antimicrobial resistance (AMR) genes in a genetically diverse group of *Salmonella enterica* recovered from foods imported into England between 2014 and 2018.

**Methods and Results:**

Whole genome sequence was used to detect AMR genes or chromosomal mutations associated with AMR in *Salmonella* recovered from edible leaves imported from Asia (*n* = 115) as compared to *Salmonella* (*n* = 231) isolated from raw chicken, 74% originated from South America. Among isolates from edible leaves, three (3%) showed resistance to at least one antimicrobial agent, two (2%) of which were multidrug resistant (MDR, resistance to three or more antimicrobial classes). Resistance to at least one antimicrobial agent was detected in 214 (93%) in the chicken isolates, with 164 (71%) showing MDR. Genetic diversity and AMR profiles were highly heterogeneous across the different serovars.

**Conclusions:**

Resistance was rare among the *Salmonella* isolates from edible leaves but common (including MDR) among those from raw chicken.

**Significance and Impact of the Study:**

Surveillance of AMR in imported foods is essential for monitoring the risk of transmission of resistance from the food chain to humans and provides added public health value to pre‐existing controls of the food chain.

## INTRODUCTION

*Salmonella* is a major cause of bacterial gastroenteritis worldwide and one of the possible routes for transmission of salmonellosis is via consumption of contaminated foods. The global burden of gastroenteritis by non‐typhoidal *Salmonella* in 2010 was estimated to cause about 94 million cases and 155,000 deaths per year, of which 85% were caused by food (Majowicz et al., 2010). Further estimates from the Global Disease Study estimated 197 million (range: 127–299 million) episodes of non‐typhoidal salmonellosis in 2016 with 84,000 deaths (range: 46,000–145,000; GBD, 2018). The economist, Jim O'Neill, has predicted that by 2050, antimicrobial resistance bacteria could cost the global economy $100 trillion a year and cause 10 million deaths a year (Taylor et al., 2014). Bacteria (including *Salmonella*) containing AMR genes or chromosomal mutations associated with AMR can be present in humans and other animals as well as the environment, all of which can provide routes to contaminate food and food ingredients. antimicrobial resistance traits can therefore be transferred between bacteria in animals and humans through the food chain, resulting in the acquisition of AMR infections (Haque et al., 2017; Marshall & Levy, 2011). This transfer of AMR from different ecological niches to humans may adversely affect the efficacy and choices of treatment available, causing infections to persist and increase the burden to healthcare systems from salmonellosis (Melnyk et al., 2015).

Approximately 40 million tonnes of food are imported into the UK each year (FSA;FSS, 2021): at the time of writing, imported foods were defined as those entering the UK from outside the European Union (EU). The UK has a series of controls in place to ensure these products meet the required safety standards. All animal products are automatically considered ‘high‐risk’ and are subject to specific import controls and border checks. Plant‐based imports of food and feed are only considered high risk if they come from certain countries where specific food or feed safety risks have been identified and need to be controlled. Within the United Kingdom Health Security Agency (UKHSA), foods are examined routinely for a range of bacterial foodborne pathogens by a network of Food Water and Environmental Microbiology Laboratories (FW&E): prior to 2020 and the COVID‐19 pandemic, approximately 10,000 tests were performed on foods each year for the detection of *Salmonella* (McLauchlin, unpublished data). The activity of examining foods includes microbiological tests performed on imported foods which are a component of the import controls in place: imported foods are sampled either at retail or through re‐enforced checks at Border Control Posts (BCPs) (as required under EU legislation (EU Regulation [EC] No. 2017/625) prior to the UK exiting the EU). Isolates of *Salmonella* from both human cases of salmonellosis and from foods are submitted to the UKHSA Gastrointestinal Bacteria Reference Unit (GBRU; Chattaway et al., 2019) where whole genome sequencing (WGS) has been implemented for the routine characterization of all isolates of *Salmonella* submitted since 2014 (Chattaway et al., 2019). WGS has the advantage of being able to not only assign a species, serovar and genotype suitable for epidemiological typing, but also to detect genetic determinants encoding antimicrobial resistance (AMR) with a high level of concordance as compared with results from phenotypic tests (Hendriksen et al., 2019; Neuert et al., 2018). These data on prevalence of AMR in food products are particularly informative when combined with data from other sources and strengthens the evidence base for the diversity and burden of AMR genes in foods: a comprehensive surveillance system of AMR in bacteria isolated from the food chain is a priority for the UK Government (HM_Government, 2019). In parts of the world where WGS or AMR monitoring may be absent or infrequent, the use of antibiotics in food production may be less well controlled which may encourage multidrug resistance (MDR: defined as resistance to three or more antimicrobial classes) developing in the food‐chain microbiota. Testing of imported foods should be part of a co‐ordinated programme to provide data to assess the risks from different types of food products. Public health risks associated with *Salmonella* contamination of imported edible betel leaves have previously been described (McLauchlin et al., 2019). We previously reported a *Salmonella* contamination rate of 16% on 279 edible leaves imported into the UK (McLauchlin et al., 2018). Further data were reported on betel leaves where a *Salmonella* contamination rate of 23% was detected among 2110 samples tested (McLauchlin et al., 2019).

For food products of animal origin such as imported chicken, the public health risk of infection is well known, and a recent international outbreak in 2021 was associated with imported chicken (ECDC; FSA, 2021). However, AMR of pathogenic bacteria from imported chicken is not routinely reported.

As part of routine surveillance of imported chicken, the UKHSA FW&E laboratories tested more than 8000 samples of raw chicken between 2014 and 2019 and the *Salmonella* contamination rate was approximately 8% (McLauchlin, unpublished data). Results from the detection of *Salmonella* in both betel leaves and raw chicken have generated multiple rapid alerts for foods and feed within the EU (https://ec.europa.eu/food/safety/rasff‐food‐and‐feed‐safety‐alerts/rasff‐portal_en).

Within the EU, risk managers respond to the results of local testing and observation of imported foods by implementing controls at the EU level and provide alerts through the Rapid Alert System for Food and Feed (RASFF) system (https://webgate.ec.europa.eu/rasff-window/portal/?event= searchResultList&StartRow = 201). Between 2011 and 2017, more than 150 RASFF notifications were generated because of *Salmonella* contamination of both betel leaves from various countries and raw chicken from Brazil. The purpose of this study was to add value to the *Salmonella* spp. isolates recovered as a result of the additional food surveillance and controls by analysing phylogenetic data and genotypic resistance profiles of this bacterium recovered from edible leaves and raw chicken imported into England.

## MATERIALS AND METHODS

### Selection of *Salmonella* isolates

Imported foods were collected as they entered the UK through BCPs, retail outlets or catering businesses by either Environmental Health Practitioners or Port Health Officers and transported in accordance with the Food Standards Agency Food Law Practice Guidance (FSA, 2017) to UKHSA FW&E microbiology laboratories located in Birmingham, London, Porton, Preston or York. All food samples were tested using the standard ISO 6579‐1 method for the detection of *Salmonella* (International Organization for Standardization, 2017). For samples collected at BCPs, five samples of 25 g from an individual consignment were tested: the definition of a consignment was defined as a ‘quantity of goods covered by the same official certificate, official attestation or any other document, conveyed by the same means of transport and coming from the same territory or third country, and being of the same type, class or description. For those samples collected at retail or catering, only a single 25 g sample was tested. Due to the fact that independent samples (usually five 25 g samples) were collected from an individual consignment inspected at the point of import, multiple *Salmonella* isolates were recovered from some consignments which were included in this study. Since products from multiple growers or farms may be included in an individual consignment, in this analysis, these were given the general term ‘batch’. For samples collected at retail, those sampled on the same day and the same retailer were considered as from the same batch.

*Salmonella* cultures isolated from foods were submitted to the UKHSA National Reference Laboratory (GBRU) for confirmation and characterization by WGS. A total of 346 *Salmonella* isolates submitted between 2014 and 2019 were selected for this study. These comprised 115 cultures from 77 batches of tropical ready‐to‐eat edible leaves (samples of 72 betel, 41 curry, one banana and one sample of pandan leaves) and 231 cultures from 115 batches of raw frozen chicken were used in this study.

### WGS and genotypic characterization of antimicrobial resistance

DNA from purified cultures of *Salmonella* was obtained by automated extraction (QIAsymphony DSP DNA Kit) according to the manufacturer's instructions (Qiagen). Genomic DNA was sequenced by the UKHSA Central Sequencing Unit: sample was prepared was using the NexteraXT (Illumina Inc) and sequenced using the Illumina HiSeq 2500 platform with 2 × 100 bp reads (Illumina Inc). Short reads were quality trimmed using Trimmomatic removing the sequence adaptor (Bolger et al., 2014). As previously described, the UKHSA KmerID pipeline (https://github.com/phe‐bioinformatics/kmerid) is used to compare the sequenced reads with published genomes to identify the bacterial species and Salmonella subspecies (Chattaway et al., 2019). The quality of the sample is further evaluated by MLST using the Achtman seven gene scheme (Achtman et al., 2012; MOST, https://github.com/phe‐bioinformatics/MOST; Tewolde et al., 2016).

Confirmation of identity of *Salmonella* spp., sequence type (ST), eBURST group (eBG), serovar and single nucleotide polymorphism (SNP) typing were derived from the genomic data as described previously (Chattaway et al., 2019; Dallman et al., 2018). In all, 33 isolates (10% of the 346 isolates) belonged to ‘uncommon’ serovars where SNP typing was not available for further analysis. The relationship between isolates within an individual eBG or ST was assessed by pairwise SNP differences: isolates with ≤5 SNPs difference from each other were considered to be indistinguishable and to have a recent common ancestor (Mook et al., 2018; Waldram, Dolan, et al., 2018a). FASTQ sequences were deposited in the National Center for Biotechnology Information Sequence Read Archive under NCBI BioProject PRJNA248792 (https://www.ncbi.nlm.nih.gov/bioproject/?term=PRJNA248792) and the reference numbers are available in Supplementary Table S1.

Antimicrobial resistance (AMR determinants were identified as described previously Neuert et al., 2018) using a customized algorithm Genefinder V1‐5 (Langmead & Salzberg, 2012; Neuert et al., 2018): AMR due to efflux pump genes was identified as described elsewhere (Braibant et al., 2005; George & Hall, 2002). MDR was defined as resistance to three or more antimicrobial classes.

## RESULTS

### *Salmonella*, countries of origin and sample setting

Of the total 346 *Salmonella* isolates from imported leaves and chicken, 99% (*n* = 344) were *Salmonella enterica, subspecies enterica* (subspecies I) and 0.6% (*n* = 2, both isolated from edible leaves) were *Salmonella enterica, subspecies houtenae* (subspecies IV; Table 1). A larger diversity of Salmonella serovars isolated from imported leaves (*n* = 33) in comparison to the imported chicken (*n* = 16) with curry leaves having the most diversity (*n* = 23; Table 1, Supplementary Table S1).

**TABLE 1:** Food types, settings where isolated and countries of origin for 346 *Salmonella enterica* isolated from edible leaves or raw chicken imported into England during 2014–2019

Food group	No. isolates tested	Food type (No. isolates)	Collection setting (No. isolates)	Countries of origin (No. isolates)	*Salmonella* serovars/subspecies identified (No. cultures)
Raw chicken	231	Frozen raw chicken (231)	Border Inspection Posts (231)	Brazil (165), Thailand (51), Chile (6), Israel (4), Not Stated (5)	*S*. Agona (11), *S*. Albany (9), *S*. Binza (1), *S*. Braenderup (4), *S*. Enteritidis (16), *S*. Give (3), *S*. Heidelberg (138), *S*. Hvittingfoss (1), *S*. Infantis (11), *S*. Kedougou (5), *S*. Mbandaka (2), *S*. Minnesota (22), *S*. Poona (2), *S*. Typhimurium (3), *S*. Virchow (1), *S*. Weltevreden (2)
Edible leaves	115	betel (72), curry (41), banana (1), pandan (1)	Border Inspection Posts (52), retail (60), catering (2), restaurant (1)	Bangladesh (21), India (20), Malaysia (11), Sri Lanka (8), Ghana (4), Dominican Republic (2), Kenya (2), Pakistan (1), Nepal (1), Thailand (1), Not Stated (44)	*S*. Aberdeen (1), *S*. Agona (1), *S. arizonae* (1), *S*. Augustenborg (3), *S*. Bareilly (9), *S*. Brunei (1), *S*. Charity (1), *S*. Cerro (1), *S. enterica* (3), *S*. Enteritidis (1), *S*. Fulica (2), *S*. Guildford (1), *S. houtenae* (2), *S*. Hvittingfoss (4), *S*. Java (2), S. Javiana (5), *S*. Kasenyi (1), *S*. Kentucky (1), *S*. Litchfield (3), *S*. Morehead (1), *S*. Mount‐pleasant (1), *S*. Newport (13), *S*. Poona (1), *S*. Rubislaw (3), *S*. Stanley (1), *S*. Stanleyville (3), *S*. Typhimurium (8), *S*. Umbilo (1), *S*. Uzaramo (4), *S*. Virchow (24), *S*. Weltevreden (6)

**FIGURE 1:**
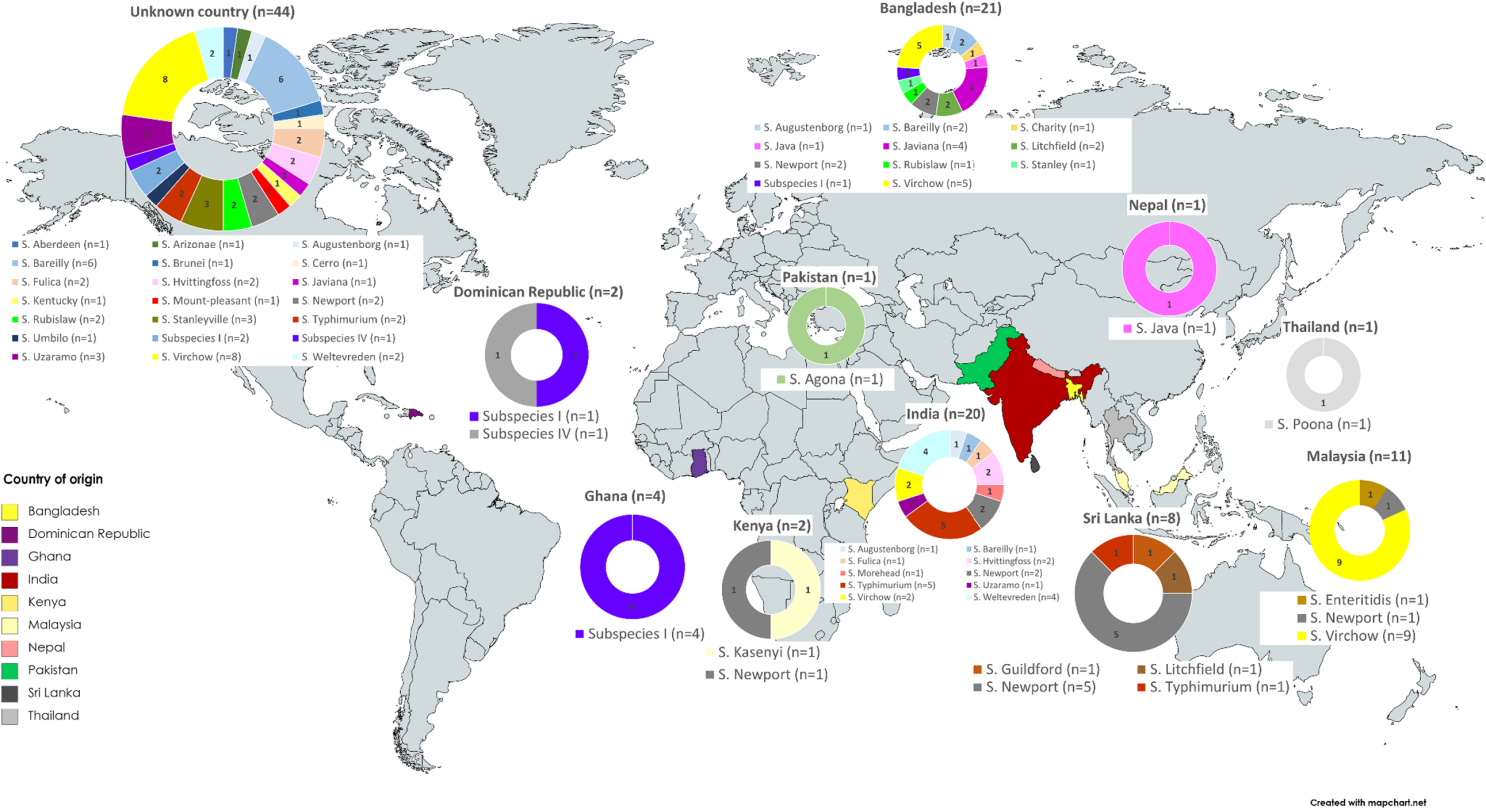
Distribution of *Salmonella* serovars recovered from imported edible leaves by country of origin during 2014–2019. World map of countries of imported leaves into England and different Salmonella serovars isolated. Figure shows that a variety of serovars were isolated from different countries and variety increased along‐side the sampling volume.

The 115 isolates from edible leaves were collected from BCPs, retail sale and from catering businesses and originated from 10 different countries of origin. Overall, 29 different serovars were identified, the most common being *S*. Virchow (*n* = 24, 20.9%), followed by *S*. Newport (*n* = 13, 11.3%) and *S*. Bareilly (*n* = 9, 7.8%; Table 1, Figure 1).

**FIGURE 2:**
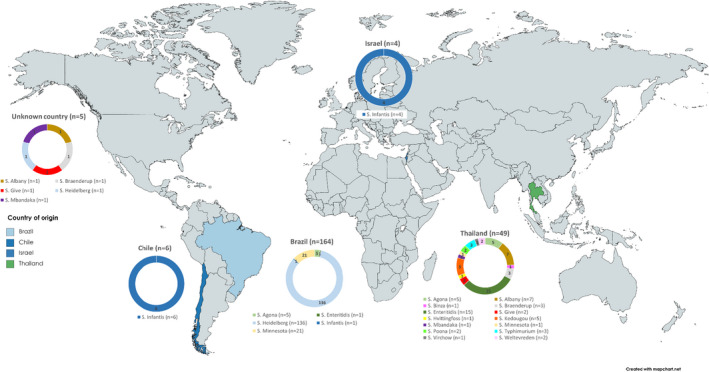
Distribution of *Salmonella* serovars recovered from imported raw chicken by country of origin during 2014–2019. World map of countries of chicken into England and different Salmonella serovars isolated. Figure shows that the diversity and presence of specific of Salmonella serovars varied depending on the country. Chicken imported from Thailand shows a variety of Salmonella serovars present, whereas chicken imported from Brazil has a predominant *S*. Heidelberg and *S*. Minnesota Salmonella population present.

All 231 isolates from raw chicken were collected from BCPs and originated from four countries of origin, most commonly from Brazil (*n* = 165, 71%) and Thailand (*n* = 51, 23%; Figure 2). A total of 16 serovars were detected, the most common being *S*. Heidelberg (*n* = 138, 60%) followed by *S*. Minnesota (*n* = 22, 9%) and *S*. Enteritidis (*n* = 16, 7%; Table 1, Figure 2). All of the 138 *S*. Heidelberg isolates were recovered from chicken originating from Brazil except for one isolate where the country of origin was not recorded. Four *Salmonella* serovars recovered from both leaves and chicken (*S*. Agona, *S*. Enteritidis, *S*. Infantis and *S*. Minnesota) were imported from more than one country of origin (Supplementary Table S1).

The diversity of *S. enterica* in relation to batch of chicken was assessed using SNP analysis. Out of the 116 batches assessed, 58 had only one isolate available and one batch had two isolates of the same serovar detected but the two isolates had not been routinely SNP typed. This left 57 batches for analysis where between 2 and 5 isolates were typed (Table 2). In 60% of batches where two or more isolates were typed, isolates fell into the same ≤5 SNPs single linkage cluster and considered as being genetically related (Mook et al., 2018; Waldram, Dolan, et al., 2018a). In 25% of batches, isolates of the same serotype fell into a 25 SNP single linkage cluster and the remaining isolates of batches fell into clusters of more than 25 SNPs. The *Salmonella* isolates from imported leaves were from a total of 77 batches and in 67 of these, due to the rarity of the serovars only one isolate had been routinely SNP typed. Of the remaining 10 batches, eight had two or more isolates with the same *Salmonella* serovar and in six of seven batches they fell into the same ≤5 SNPs cluster.

**TABLE 2:** Diversity of *Salmonella* recovered from the individual chicken meat batches imported into England during 2014–2019

Diversity of salmonella isolates	Number of batches with				Total number of batches where ≥2 isolates^a^	% of batches with two or more isolates typed for each diversity level indicated
	2 isolates	3 isolates	4 isolates	5 isolates		
0–5 SNPs (same serovar)	18	3	7	6	34	59.6
6–25 SNPs (same serovar)	3	1	0	0	4	7.0
>25 SNPs (same serovar)	3	5	5	1	14	24.6
2 or more serovars	4	0	1	0	5	8.8
**Total**	29	9	13	7	57	100

Abbreviations: NA, not applicable; SNP, single nucleotide polymorphism; SLC, single linkage cluster.

^a^ In total, isolates from 116 batches were analysed where 57 batches had two or more isolates with SNP typing available.

### Detection of genetic determinants for AMR

#### Imported leaves

Out of the 115 *S*. enterica isolated from edible leaves, AMR determinant markers were detected in only three (3%) *Salmonella* isolates and these were recovered from three curry leaf samples. One *S*. Weltevreden isolate (381344), imported from India in 2017, exhibited a double mutation in gyrA[87:D‐N]/parC[57:T‐S] conferring resistance to fluoroquinolones. Another *S*. Weltevreden isolate (412029) from an unnamed country in 2017 harboured resistance genes to ampicillin (blaTEM‐1), azitrhomycin (mphA), trimethoprim (*dfrA12*), tetracycline (*tet*M, *tet*A), sulphonamide (*sul1, sul3*), chloramphenicol (*catA*) and a plasmid‐mediated quinolone‐resistant (PMQR) determinant conferring resistance to fluoroquinolone (*qnrS1*). Finally, a *S*. Agona isolate (31140780), imported from Pakistan, harboured resistance genes to trimethoprim (*dfrA5*), tetracycline (*tet*A) and sulphonamide (*sul1*) (Supplementary Table S1).

#### Imported chicken

**FIGURE 3:**
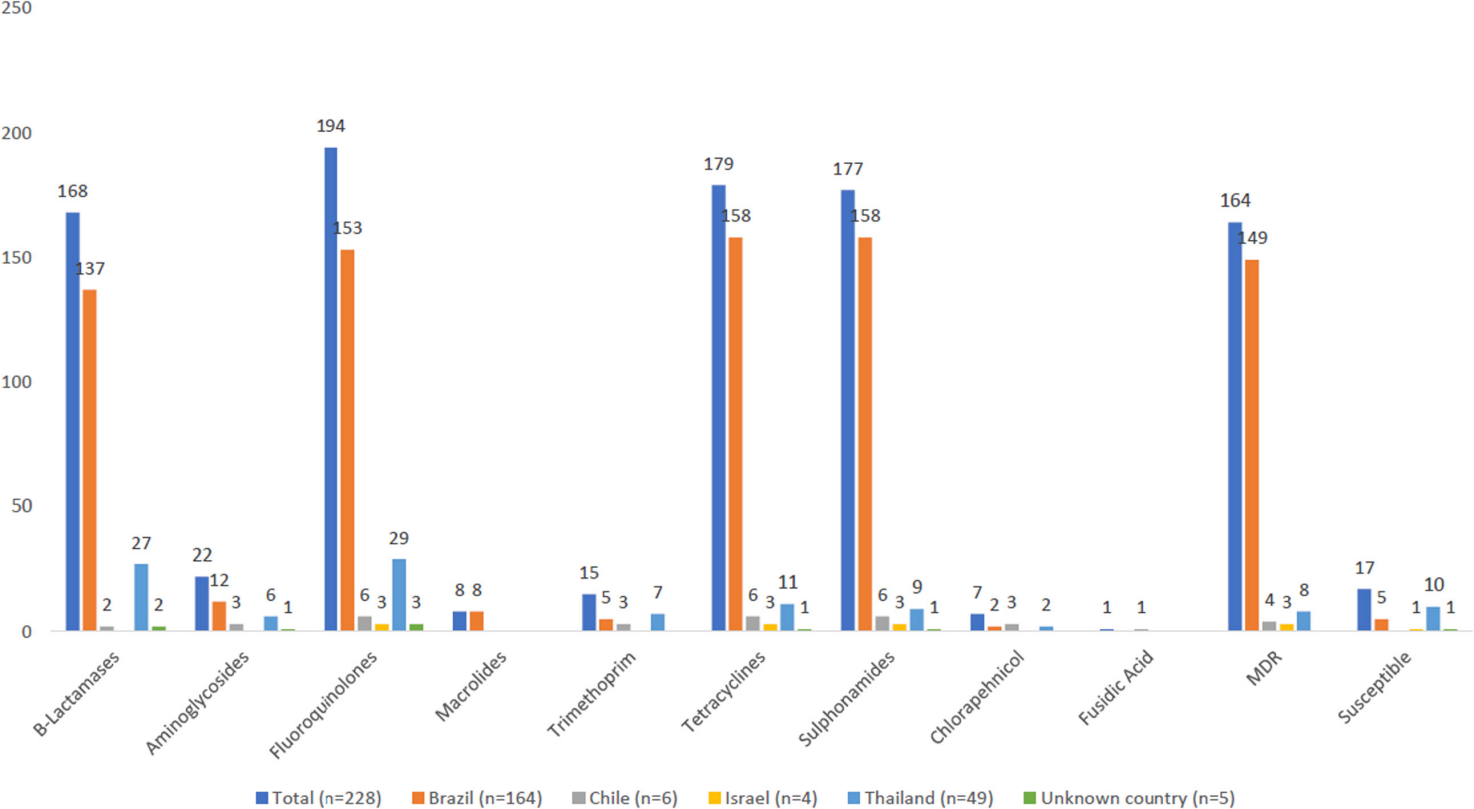
antimicrobial resistance determinants of *Salmonella* isolated from chicken by country of import during 2014–2019. Bar graph showing the presence of antimicrobial resistance determinants in Salmonella isolate from imported chicken. Figures shows that isolates were more likely to be associated with antimicrobial resistance rather than susceptible with a large majority having multiple drug resistance.

Among the 231 *Salmonella* spp. isolated from chicken, 214 (93%) had at least one mutation or resistance gene determinant, the most common being to β‐Lactams, fluoroquinolones, tetracyclines or sulphonamides (Figure 3, Table 3): only 17 (7%) had no resistance genes detected. Seventy‐one percent of the chicken isolates (*n* = 164) were MDR and these were recovered from products imported from all four countries of origin (Figure 3; Table 3, Supplementary Table S1).

**TABLE 3:** AMR genotypic combinations in Salmonella serovars recovered from imported chicken meat batches imported into England during 2014–2019

Serovar (No. isolates), country year	Number of isolates (number of batches)	Genotypic combinations
***S*. Heidelberg (138)**		
Brazil 2017, 2018	95 (49)	*bla*_CMY‐2_, *sul2*, *tetA*, *gyrA*(83:SF), *parC*(57:TS), *aac(6)‐Iy*
Brazil 2017	2 (1)	*bla*_CMY‐2_, *sul2*, *tetA*, *gyrA*(83:SF), *parC*(57:TS), *dfrA1*, *aac(6)‐Iy*
Brazil 2017	2 (2)	*bla*_CMY‐2_,*sul1*, *sul2*, *tetA*, *gyrA*(83:SF); *parC*(57:TS), *aac(6)‐Iy*, aac(3)‐Via, aadA‐1
Brazil 2017	1	*bla*_CMY‐2_,*sul2*, *tetA*, *gyrA*(83:SF), *parC*(57:TS), *qnr*B19, *aac(6)‐Iy*
Brazil 2017	2 (2)	*bla*_CMY‐2_,*sul2*, *tetA*, *gyrA*(83:SF), *parC*(57:TS), *dfrA1*, *floR*, *aac(6)‐Iy*, *aph(6)‐Id*, *strA*, *strB*
Brazil 2017	1	*bla*_CMY‐2_,*sul2*, *tetA*, *gyrA*(83:SF), *parC*(57:TS), *aac(6)‐Iy*, *aph(6)‐Id*, *strA*, *strB*
Brazil 2017, 2018	6 (2)	*bla*_CTX‐M‐2_,*sul1*, *sul2*, *tetA*, *gyrA*(83:SF), *parC*(57:TS), *aac(6)‐Iy*, aac(3)‐Via, aadA1
Brazil and country unknown 2017	2 (2)	*bla*_CTX‐M‐8_,*sul2*, *tetA*, *gyrA*(83:SF), *parC*(57:TS), *aac(6)‐Iy*
Brazil 2017	1	*bla*_CTX‐M‐2_, *bla*_TEM‐1_, *sul1*, *sul2*, *tetA*, *gyrA*(83:SF), *parC*(57:TS), *aac(6)‐Iy*, aac(3)‐Via, *aph(6)‐Id*, *strA*, *strB*, mphB
Brazil 2017	1	*bla*_CTX‐M‐2_, *bla*_CTX‐M‐55_, *sul1*, *sul2*, *tetA*, *gyrA*(83:SF), *parC*(57:TS), *aac(6)‐Iy*, aac(3)‐Via, aadA‐1
Brazil 2017	1	*bla*_CMY‐54_, *sul2*, *tetA*, *gyrA*(83:S‐F), *parC*(57:T‐S), *aac(6)‐Iy*
Brazil 2017	2 (2)	*bla*_TEM‐1_, *sul1*, *sul2*, *tetA*, *gyrA*(83:SF), *parC*(57:TS), aac(3)‐Via, *aac(6)‐Iy*, *strA*, *strB*, mphB
Brazil 2017	1	*bla*_BIL‐1‐_, *sul2*, *tetA*, *gyrA*(83:S‐F); *parC*(57:T‐S), *aac(6)‐Iy*
Brazil 2017	12 (8)	*sul2*, *tetA*, *gyrA*(83:SF), *parC*(57:TS), *aac(6)‐Iy*
Brazil 2017	5 (1)	*sul2*, *tetA*, *gyrA*(83:SF), *parC*(57:TS), *aac(6)‐Iy*, mphB
Brazil 2018	3 (1)	*sul2*, *tetA*, *gyrA*(83:SF), *parC*(57:TS), *qnr*B19, *aac(6)‐Iy*
***S*. Minnesota (22)**		
Brazil 2017, 2018	8 (5)	*bla*_CMY‐2_, *sul2*, *tetA*, *parC*(57:TS), *qnr*B19, *aac(6)‐Iy*, aadA1b
Brazil 2017, 2018	6 (5)	*bla*_CMY‐2_, *sul2*, *tetA*, *parC*(57:TS), *qnr*B19, *aac(6)‐Iy*
Brazil 2017	5 (1)	*bla*_CMY‐2_, *sul2*, *tetA*, *parC*(57:TS), *aac(6)‐Iy*, aadA1b
Brazil 2018	1	*bla*_CMY‐2_, *sul2*, *tetA*, *parC*(57:TS), *aac(6)‐Iy*
Brazil 2017	1	*bla*_CMY‐2_, *sul2*, *tetA*, *parC*(57:TS), *qnr*B19, *aac(6)‐Iy*
Thailand 2017	1	*bla*_CMY‐2_, *bla*_CTX‐M‐8_, *sul2*, *tetA*, *parC*(57:TS), *qnr*B19
***S*. Enteritidis (16)**		
Thailand 2015, 17, 18	11 (6)	*bla*_TEM‐135_, *gyrA*(87:DY), *aac(6)‐Iy*
Thailand 2016, 17	4 (3)	*gyrA*(87:DY), *aac(6)‐Iy*
Brazil 2017	1	*gyrA*(87:DN), *aac(6)‐Iy*
***S*. Infantis (11)**		
Chile 2018	2 (1)	*bla*_CTX‐M‐65_, *sul1*, *tetA*, *gyrA*(87:DY), *parC*(57:TS), *floR*, *aac(6)‐Iy*, *aac(3)‐Iva*, *aadD*, *aph(4)‐Ia*,
Chile 2018 Brazil 2017	3 (2) 1	*sul1*, *tetA*, *gyrA*(87:DY), *parC*(57:TS), *dfrA1*4, *aac(6)‐Iy*
Israel 2015	3 (1)	*sul1*, *tetA*, *gyrA*(87:DY), *parC*(57:TS), *aac(6)‐Iy*
Chile 2018	1	*sul1*, *tetA*, *gyrA*(87:DY), *parC*(57:TS), *floR*, *fosA3*, *aac(6)‐Iy*, *aac(3)‐Iva*, *aadD*, *aph(4)‐Ia*
Israel 2015	1	*parC*(57:TS), *aac(6)‐Iy*
***S*. Agona (11)**		
Thailand 2017	5 (2)	*bla*_TEM‐1_, *sul3*, *tetA*, *parC*(57:TS), *qnr*S1, *dfrA1*4, *aac(6)‐Iy*
Brazil 2017	5 (1)	*parC*(57:TS), *aac(6)‐Iy*
Thailand 2014	1	*bla*_TEM‐117_, *parC*(57:TS), *lnuF*, *aac(6)‐Iy*, aac(3)‐IId, *aadA2*, *aadA17*
***S*. Albany (9)**		
Thailand 2014 Unknown 2017	5 (3) 1	*bla*_TEM‐117_, *parC*(57:TS), *aac(6)‐Iaa*, aac(3)‐IId, *aadA2*, *aadA17*, *lnuF*
Thailand 2015, 2016	2 (2)	*bla*_TEM‐117_, *parC*(57:TS), *aac(6)‐Iaa*, *aadA2*, *aadA17*, *lnuF*
Thailand 2015	1	*bla*_CARB‐2_, *sul1*, tet(G), *gyrA*(87:DN), *parC*(57:TS), *dfrA1*, *floR*, *aac(6)‐Iaa*
***S*. Kedougou (5)**		
Thailand 2017	5 (1)	*aac(6)‐Iaa*
***S*. Braenderup (4)**		
Thailand and unknown 2017	4 (2)	*gyrA*(87:D‐Y), *parC*(57:TS), *aac(6)‐Iaa*
***S*. Give (3)**		
Thailand 2015, 2017 Unknown 2017	2 (2) 1	*gyrA*(83:S‐Y), *parC*(57:TS), *aac(6)‐Iy*
***S*. Typhimurium (3)**		
Thailand 2014	1	*bla*_TEM‐191_, *sul2*, *tetA*, *aac(6)‐Iaa*, aac(3)‐IId, *aadA2*, *aadA17*, *aph(6)‐Id*, *strB*, *strA*, *lnuF*,
Thailand 2016	1	*bla*_TEM‐117_, *aac(6)‐Iaa*, aac(3)‐IId, *aadA2*, *aadA17*, *lnuF*
Thailand 2016	1	*aac(6)‐Iaa*
***S*. Mbandaka (2)**		
Thailand and unknown country 2017	2 (2)	*parC*(57:TS), *aac(6)‐Iaa*
***S*. Poona (2)**		
Thailand 2016	2 (1)	*parC*(57:TS), *aac(6)‐Iaa*
***S*. Weltevreden (2)**		
Thailand 2017	2 (1)	*tetA*, *parC*(57:TS), *aac(6)‐Iaa*
***S*. Binza (1)**		
Thailand 2015	1	*bla*_TEM‐1_, *sul3*, *tetA*, *gyrA*(83:SY), *parC*(57:TS), *dfrA1*2, cml1, *aac(6)‐Iy*, *aadA2*, *aadA12*, *lnuF*, mefB
***S*. Hvittingfoss (1)**		
Thailand 2017	1	*aac(6)‐Iaa*
***S*. Virchow (1)**		
Thailand 2015	1	*gyrA*(87:DY), *aac(6)‐Iy*

All *S*. Heidelberg isolates had resistance genes for fluoroquinolones, tetracyclines and sulphonamides and detected in 2017 and 2018 showing a recent emergence of the strain at that time. Out of the 138 *S*. Heidelberg isolates, 120 (87%) were isolated during examination of chickens in 2017 with only 18 (13%) being isolated in 2018. Five isolates from 2017 (4%) had *bla*_CTX‐M_ markers associated with cephalosporin resistance with genes including *bla*_CTX‐M‐2_, *bla*_CTX‐M‐8_ and *bla*_CTX‐M‐55_, while five (28%) isolates from 2018 contained CTX‐M genes (*bla*_CTX‐M‐2_). None of the 2018 isolates had resistance genes for macrolides (*mphB*), trimethoprim (*dfrA1*) or chloramphenicol (*floR*), compared to isolates collected in 2017 containing *mphB* genes (*n* = 8, 44%), *dfrA1* genes (*n* = 4, 22%) and *floR* genes (*n* = 2, 11%). A diversity of resistance genes for aminoglycosides (different combinations of aac(6′)‐Iy, aac(3)‐VIa;aph(6)‐Id, aadA1,strA‐strB) and sulphonamides (*sul1, sul2*) were present in seven (6%) 2017 isolates and five (28%) 2018 isolates. There was an increase in *qnrB19* genes (fluoroquinolones) from one (0.8%) in 2017 to three (17%) in 2018 (Table 3, Supplementary Table S1).

The resistance profile for five of six *S*. Agona isolates from Thailand recovered from two batches (97 and 98) showed resistance to five classes of antibiotics (β‐Lactams, fluoroquinolones, trimethoprim, tetracyclines and sulphonamides) while *S*. Agona isolates from a single batch from Brazil (*n* = 5) all had no resistance genes (Table 3, Supplementary Table S1).

The resistance profile of *S*. Infantis isolates varied; Isolates from Brazil, Chile and Israel (*n* = 1, *n* = 6 and *n* = 3, respectively) shared the same resistance determinants for fluoroquinolones (*gyrA*[87:D‐Y];*parC*[57:T‐S]), tetracyclines (*tetA*) and sulphonamides (*sul1*). In contrast, *S*. Infantis isolates from Chile and Israel differed in AMR profiles including additional resistance genes for trimethoprim (*dfrA14*), extended spectrum β‐Lactamase (ESBL; *bla*_CTX‐M‐65_), chloramphenicol (*floR*) and fusidic acid (*fosA3*) (Table 3, Supplementary Table S1).

All *S*. Enteritidis isolates from Thailand (*n* = 15/231, 6%) had a single mutation in *gyrA*[87:D‐Y] conferring resistance to fluoroquinolone and 10 of these isolates had additional the β‐Lactamase resistance gene *bla*_TEM‐135_. The single *S*. Enteritidis isolate (370886) from Brazil had the same single mutation in *gyrA*[87:D‐N]. *S*. Minnesota isolates from Brazil (*n* = 21, 9%) and Thailand (*n* = 1, 0.4%) had similar resistance profiles including resistance genes for tetracyclines and sulphonamides, with an additional ESBL resistance gene (*bla*_CTX‐M‐8_) in the isolate (357771) from Thailand (Table 3, Supplementary Table S1).

### Resistance to β‐lactams

A total of 170 (74%) of the isolates from chicken had determinants conferring resistance to β‐lactam antibiotics with the plasmid encoded *bla*_CMY‐2_ (*n* = 124, 54%) being the most common. Penicillinase‐encoding β‐lactam genes were detected in 30 (13.0%) isolates including *bla*_TEM‐135_ (*n* = 11, 5%), *bla*_TEM‐1_ (*n* = 9, 4%), *bla*_TEM‐117_ (*n* = 8, 3%) and *bla*_TEM‐191_ (*n* = 1, 0.4%). Genes for *bla*_CTX‐M_ type ESBLs were present in 13 (6%) isolates, including *bla*_CTX‐M‐8_ (*n* = 3, including two *S*. Heidelberg 1%, 1 *S*. Minnesota, 0.4%), *bla*_CTX‐M‐2_ (*n* = 8 *S*. Heidelberg, 3%), *bla*_CTX‐M‐65_ (*n* = 2 *S*. Infantis, 1%) and *bla*_CTX‐M‐55_ (*n* = 1 *S*. Heidelberg, 0.4%). Three of these isolates had a combination of these genes: *bla*_CTX‐M‐55_, *bla*_CTX‐M‐2_; *bla*_CTX‐M‐2_, *bla*_TEM‐1_ and *bla*_CMY‐2_, *bla*_CTX‐M‐8_ (Table 3, Supplementary Table S1).

Isolates with identical SNPs addresses had different AMR genes detected, and included two S. Heidelberg isolates from the same batch of Brazilian chicken (385,530 and 385,529) had different genes for β‐lactam resistance (*bla*_BIL‐1_ or *bla*_CMY‐2_) but had the same SNP address; the same SNP was also detected for two isolates (440,917 and 440,919) from different batches that harboured *bla*_CMY‐2_ or *bla*_CMY‐54_ (batches 92 and 93, respectively) (Table 3, Supplementary Table S1).

### Resistance to quinolones

A total of 222 (96%) of the isolates from chicken had at least one mutation in the quinolone resistance‐determining region (QRDR). Different mutations for QRDRs of the DNA gyrase subunit gene *gyrA* in combination with multiple mutations in the DNA topoisomerase gene *parC* were observed in 158 (68%) isolates, the most common being *gyrA*[83:S‐F]/*parC*[57:T‐S] (*n* = 134, 58.0%). A further 41 isolates (18%) harboured determinants expected to confer reduced susceptibility to ciprofloxacin with or without *parC* mutations. These included a single *gyrA* mutation in the QRDR (*n* = 17, 7%), the most common being *gyrA*[87:D‐Y] (*n* = 16, 7%) and/or one or multiple PMQR genes (*n* = 25, 11%). The most frequent PMQR genes detected were *qnrB19* (*n* = 20, 9%) and *qnrS1* (*n* = 5, 2%). There were 27 (12%) isolates with only a *parC* mutation, although this is not known to confer resistance to fluroquinolone (Neuert et al., 2018; Table 3, Supplementary Table S1).

The presence of Aminoglycosides Genes predicted to confer resistance to streptomycin (*strA*, *n* = 1, 0.4% and *strB*, *n* = 7, 3%), were detected in seven isolates and 38 (16%) isolates carried genes encoding aminoglycoside adenyltransferase, most commonly *aadA1b* (*n* = 14, 6.%). All 231 isolates carried an aminoglycoside acetyltransferase *aac*(*6′*)‐type gene. However, the majority had the *aac*(*6′*)‐*Iy* (*n* = 203, 88%), followed by *aac(6′)‐Iaa* (*n* = 28, 12%; Table 3, Supplementary Table S1).

Aminoglycoside acetyltransferase *aac(3)* variants associated with resistance to gentamicin and tobramycin (Neuert et al., 2018) were detected in 25 isolates from five serovars. Most notably *aac(3)‐IId* (*n* = 7, 3.%) and *aac(3)‐IVa* (*n* = 3, 1%). *Aac(3)‐*VIa, which confers resistance to the veterinary aminoglycoside apramycin (Neuert et al., 2018), was present in 12 isolates (5%). No *aac(3)* variants were found in 206 (89%) *Salmonella* isolates. Furthermore, the aminoglycoside phosphotransferase genes *aph(4)‐Ia* (*n* = 3, 1%) and *aph(6)‐Id* (*n* = 7, 3%) were identified. No 16S rRNA methyltransferase genes were detected (Table 3, Supplementary Table S1).

### Resistance to sulphonamides, tetracyclines, trimethoprim and chloramphenicol

Sulphonamide resistance genes were found in 178 isolates (77%): 161 (70%) carried *sul2*, 23 (10%) carried *sul1*, 12 (6%) isolates carried both and 6 (3%) carried *sul3* (Supplementary Table S1). Tetracycline resistance genes occurred in 180 isolates (78%), most commonly *tet*A (*n* = 179, 77%). An additional, less frequently encountered gene was the efflux pump‐encoding *tet*G (*n* = 1, 0.4%). Trimethoprim resistance conferring *dfrA* gene variants were identified in 15 (6%) isolates: *dfrA14* (*n* = 9, 4%), *dfrA1* (*n* = 5, 2%) and *dfrA12* (*n* = 1, 0.4%; Table 3, Supplementary Table S1).

Genes linked to chloramphenicol resistance were identified in seven isolates. Efflux pump genes were found in six isolates: *floR* (*n* = 6, 3%) or *cml1* (*n* = 1, 0.4%; Table 3, Supplementary Table S1).

## DISCUSSION

antimicrobial resistance (AMR) is an increasing threat to public health and sustainable development (WHO, 2020). The evaluation of the use of the genome sequence for AMR prediction has been reviewed (Hendriksen et al., 2019) and the application of this technique for global surveillance can provide information on the early emergence and spread of AMR and further inform timely policy development on AMR control. Sequencing data emanating from AMR surveillance provides key information to guide the development of rapid diagnostic tools for better and more rapid characterization of AMR, and thus complement phenotypic methods. Although not without limitations and cannot totally replace phenotypic methods, analysis by WGS provides information on determinants known to confer antimicrobial resistance. This study used WGS to provide data on the distribution of AMR genes among 343 *Salmonella* isolates from two different types of imported food products, that is, ready‐to‐eat edible leaves and raw chicken. Previous results from this laboratory showed that the WGS strategy utilized here generated highly accurate prediction of AMR between results from WGS and phenotypic tests using non‐typhoidal *Salmonella* from human clinical infections (Hendriksen et al., 2019; Neuert et al., 2018). We also found a high correlation between the analysis of WGS data to deduce AMR and results of phenotypic testing and will publish these data elsewhere.

The study utilized Salmonella comprising 39 serovars, of which 30 different serovars were from edible leaves and 16 were isolated from chicken. The specific ready‐to‐eat food of edible betel leaves produced outside of the EU have previously been identified as being particularly commonly contaminated by enteric bacteria (McLauchlin et al., 2019). Fresh basil from Israel was associated with a *S*. Senftenberg outbreak (Elviss et al., 2009; Pezzoli et al., 2008). Fruit and vegetables, particularly those which are more prone to bacterial contamination and are usually consumed raw, were identified as a priority by the European Food Safety Authority with respect to the monitoring for carbapenamase‐producing bacteria (EFSA BIOHAZ Panel, 2013). However, there is comparatively little information on the prevalence of AMR among *Salmonella* recovered from edible leaves and in this study, AMR was rare: 97% of isolates from leaves had a genotypic profile in line with the typical intrinsic resistance to aminoglycosides in *Salmonella*. These results contrast with that reported by Singh et al. who investigated 120 *Salmonella* isolates from betel leaves and associated ‘soaking waters’ in Northern India: all isolates were resistant to at least one of the antimicrobial agents tested and four were MDR (Singh et al., 2006). In a second study, only one out of 25 *Salmonella* isolates from betel leaves collected in Bangladesh tested were resistant to ampicillin, chloramphenicol and cephalexin (Haque et al., 2017). These contrasting studies could be interpreted that the country of origin has a role in the resistance of organisms with those from leaves in this study showing a low level of resistance irrespective of the country origin. It is intriguing that among the small number of resistant organisms from leaves, one was a *S*. Agona (31140780) that was associated with a UK outbreak linked to curry leaves produced in Pakistan (Waldram, Lawler, et al., 2018b). Since this outbreak, the *S*. Agona MDR strain was sporadically detected up to 2019 in the UK, the majority of isolates from human faeces from individuals with a travel history to Pakistan (data not shown). This indicates that this *S*. Agona is likely to be an endemic strain in Pakistan that has the ability to enter to food chain and highlights the importance of monitoring imported food products alongside clinical cases.

The use of antimicrobial agents as growth promoters and for prophylactic or therapeutic treatment in food animal production is well recognized and concerns raised if this would encourage AMR bacteria to enter the food chain. While this has been tightly controlled within the EU since 2006, more relaxed controls may occur in other countries (Willis et al., 2018). Imported raw chicken has been previously identified as a source of AMR in bacteria (Verraes et al., 2013) and this study sought to understand if this was occurring in England. The most frequent serovar detected in imported chicken detected in this study was *S*. Heidelberg, from Brazil and were resistant to three or more antimicrobial classes (Tables 1, 3). This finding supports the alerts in the RASFF (https://ec.europa.eu/food/safety/rasff‐food‐and‐feed‐safety‐alerts/rasff‐portal_en) regarding *Salmonella* contamination of chicken production in Brazil, with additional checks on Brazilian chicken being required at EU BCPs. A high proportion of MDR in S. Heidelberg isolates has also been previously reported in isolates recovered from poultry imported into the Netherlands from South America, including Brazil, where all 119 *S*. Heidelberg isolates examined between 2010 and 2015 showed 96% (*n* = 158) of isolates in chicken from Brazil had MDR, of which 82% (n = 134) had ESBL markers (van den Berg et al., 2019). However, there was a greater variety in the β‐lactam resistance genes compared to those imported to the Netherlands highlighting that there may be country‐specific clones responsible for MDR expansion rather than the serovar itself (van den Berg et al., 2019). To test this hypothesis, a subset of the isolates tested in this study was further analysed: Alikhan et al confirmed the AMR results described here and further showed that the S. Heidelberg cultures form clades distinct from global isolates, with temporal analysis suggesting emergence of this serovar in the early 2000s, around the time of the 2003 introduction of the *S*. Enteritidis vaccine into Brazilian poultry production (Alikhan et al., 2022).

In contrast to chicken production, it is less likely that antimicrobials would be used as part of the farming process for edible leaves. This may account for the larger diversity of Salmonella serovars found in imported leaves in comparison to the imported chicken in this study. Although antimicrobial agents such as streptomycin have been used as pesticides, there are fewer benefits to the use of antimicrobials in horticulture. Salmonellas recovered from leaves are likely to be as a result of exposure to irrigation or wash water contaminated with human, domestic or wild animal's faeces or through contact with contaminated equipment or food handlers. In addition, exposure to antimicrobial agents, environmental factors also play a key role in Salmonella colonization of food products and may account for the different diversity of serovars in the different edible leaves. Studies have shown that that organic fertilizers can increase the persistence of Salmonella in soil and that soil type and plant species play a crucial role in the interactions between human pathogens and crop plants (Jechalke et al., 2019). The low level of resistance among the *Salmonella* contaminating the edible leaves indicates that there is either limited exposure to antibiotics in the local animal or human populations from which the contaminants originated or that the drivers for selection and retention of resistance do not occur in the same way as in poultry production.

Chicken can be exposed to *Salmonella* via a variety of routes including contaminated feed, bedding, soil and faecal matter as well as from vermin such as rodents acting as vectors. Chicken are also capable of passing strains via horizontal and vertical transmission both within and between flocks (Andino & Hanning, 2015). *Salmonella* can be transferred into food and food ingredients during slaughter and processing as well as via cross‐contamination prior to consumption. Consumption of chicken, predominately undercooked chicken, is a risk factor for acquiring salmonellosis in humans. An increase in *Salmonella* outbreaks associated with minimally processed foods such as salads and herbs has occurred since the early 2000s (Schierstaedt et al., 2019), as well as a large outbreak associated with the consumption of curry leaves (Waldram, Lawler, et al., 2018b), has increased interest in edible leaves as a food safety risk. The occurrence of *Salmonella* in edible imported herbs and leaves in the human food chain in England was previously reported (McLauchlin et al., 2018; Willis et al., 2015). Contamination is most likely to occur at primary production (during cultivation, from contaminated soil or water, by contact with livestock or human waste or from wild animals) but may also occur through poor hygiene throughout the food chain (Schierstaedt et al., 2019).

There is little information published about the genetic diversity of *Salmonella* within consignments of imported products. In this study, where *Salmonella* was detected, 60% of consignments were contaminated with the same genetically related strain (within a 5 SNP cluster) and showed an identical AMR profile indicating exposure to the same source of contamination. However, the other consignments showed a greater diversity with either different Salmonella serovars or more genetically distinct strains within the same serovar with different AMR profiles. This would indicate contamination from an endemic source where the population has diversified or where multiple different contamination events had occurred. This study has highlighted the complexity of *Salmonella* contamination in different food products and enables an understanding of the diversity of salmonellae and their AMR profiles.

We have previously assessed the public health risks from edible leaves and raw chicken. Betel leaves are a ready‐to‐eat food which is frequently contaminated with *Salmonella*. Although two cases (2015 and 2017) were identified as infected by the same type as that contaminating betel leaves, because of the extreme diversity of the *Salmonella* types contaminating this food, the chance of identifying the same types of this bacterium infecting humans will be very rare (McLauchlin et al., 2019). Furthermore, we also reported that the types of *Salmonella* from Brazilian raw chicken corresponded to ≤0.5% of the human cases of salmonellosis in the UK between 2004 and 2019 (Alikhan et al., 2022). However, among MDR *Salmonella* from edible leaves was a *S*. Agona from curry leaves produced in Pakistan which was associated with a large multi‐agent outbreak of gastroenteritis in 2013 (Waldram, Lawler, et al., 2018b). Of 1336 *S*. Agona isolates from cases of salmonellosis in the UK between 2013 and 2022, 10 were from the 2013 outbreak and a further 18 cases occurred where isolates were within 5 SNPs of all the cultures from the outbreak and from the curry leaves and occurred across multiple regions of England and Wales between 2014 and 2019: a travel history was available in 12 patients, 11 of which was to Pakistan (a travel history was available for 445 of the remaining patients infected by S. Agona, of which 121 was to Pakistan). Of the *S*. Agona from the 18 cases occurring between 2014 and 2019 and infected by the outbreak strain, all had the same antibiotic resistance determinants as that detected in the isolate from curry leaves, that is, resistance to aminoglycosides (aac(6′)‐Iy[v]), fluoroquinolones parC(57:TS), trimethoprim (*dfrA5*), tetracycline (*tet*A), sulphonamide (*sul1*), with the exception that all had a gyrA_(SET[83:S‐Y]) mutation conferring resistance to fluoroquinolones and one clinical isolate did not have the sulphonamide resistance gene. This observation illustrates the need to combine epidemiological, microbiological and AMR data from both human and food surveillance activities for both disease and food control purposes. Routine testing of bacterial isolates from food products via genomic sequencing will enable real‐time monitoring of AMR genes entering the food chain as well as the detection of contamination of *Salmonella* posing a risk to health. Further analysis is needed to compare data with clinical cases to assess the impact of imported AMR in isolates from food and support risk assessment in food safety and outbreak investigation.

In conclusion, we report here marked differences in the presence of AMR resistance genes or chromosomal mutations associated with AMR in *Salmonella* between those isolated from imported edible leaves and raw chicken. Detecting AMR is essential for monitoring the risk of transmission of resistance from the food chain to humans. Testing *Salmonella* isolates from imported foods provides added value to pre‐existing data and contributes to monitoring of the food chain.

## FUNDING INFORMATION

This study was funded by UKHSA and University College London. Marie Anne Chattaway is affiliated to the National Institute for Health Research Health Protection Research Unit (NIHR HPRU) in Genomics and Enabling Data at University of Warwick in partnership with the UK Health Security Agency (UKHSA), in collaboration with University or Cambridge and Oxford. MAC is based at UKHSA. The views expressed are those of the author(s) and not necessarily those of the NIHR, the Department of Health and Social Care or the UK Health Security Agency.

## CONFLICT OF INTEREST

No conflict of interest declared.

## CONSENT FOR PUBLICATION

This study does not contain individual person's data in any form.

## Supporting information


Table S1
